# Serostatus and Epidemiological Characteristics for Atypical Pneumonia Causative Bacteria among Healthy Individuals in Medina, Saudi Arabia, a Retrospective Study

**DOI:** 10.3390/healthcare10112316

**Published:** 2022-11-18

**Authors:** Sari T. Alhoufie, Areej A. Alhhazmi, Waleed H. Mahallawi, Khalid O. Alfarouk, Nadir A. Ibrahim

**Affiliations:** 1 Medical Laboratories Technology Department, College of Applied Medical Sciences, Taibah University, Al-Madinah Al-Munwarah 42353, Saudi Arabia; 2 Zamzam Research Center, Zamzam Medical College, Khartoum 11123, Sudan; 3 Biomedical Research LLC, Temple Terrace, FL 33617, USA

**Keywords:** *Chlamydia pneumoniae*, community-acquired atypical pneumonia, *Legionella pneumophila*, *Mycoplasma pneumoniae*

## Abstract

**Background:** Community-acquired atypical pneumonia is generally a mild and self-limiting infection. Still, it may lead to hospitalization and progressive clinical complications in some cases, particularly among the elderly and individuals with chronic diseases. *Chlamydia pneumoniae*, *Legionella pneumophila,* and *Mycoplasma pneumoniae* are the community’s main causative agents of atypical pneumonia. However, most published studies evaluated their incidence in the hospital setting, and little is known about their prevalence among healthy individuals. This work aims to assess the seroprevalence of these bacteria among healthy people to determine the status of immunity against these bacteria in the community. **Methodology:** Two hundred and eighty-three serum samples from a multicenter in Medina, Saudi Arabia, were collected in this study. Serum samples were subjected to indirect enzyme-linked immunosorbent assays (ELISAs) to detect IgG antibodies against *C. pneumoniae*, *L. pneumophila,* and *M. pneumoniae* to investigate the seroprevalence of these bacteria and their distribution among different genders and age groups of healthy people. **Results:** IgG seropositivity for at least one of the three atypical pneumonia-causative bacteria occurred in 85.8% (n= 243/283) of the sample population. IgG seropositivity for *C. pneumoniae* occurred in 80.6% (228/283) of the population, followed by 37.5% for *L. pneumophila* and 23% for *M. pneumoniae* (66/283). In addition, the IgG seropositivity rates for the three bacteria were observed predominantly among male participants. Furthermore, no significant difference in IgG seropositivity distribution occurred between different age groups of healthy people for *C. pneumoniae, L. pneumophila* and *M. pneumoniae*. **Conclusions:** The current study found that *C. pneumoniae*, *L. pneumophila,* and *M. pneumoniae* tended to be highly prevalent among healthy people and more common among males than females. Additionally, their pattern of distribution among healthy individuals seemed to be predominant among young adults (aged 20–40 years), which differs from their predominant distribution among elderly patients in hospital settings (>50 years).

## 1. Introduction

Pneumonia is an infection of the lower respiratory tract, particularly the alveolar space, and is associated with high morbidity and mortality [1]. Pneumonia can lead to respiratory complications such as acute respiratory distress syndrome (ARDS), abscess, and parapneumonic effusion or non-respiratory complications, including neurological and cardiac complications as well as septic shock and sepsis [2]. The most common symptoms of pneumonia are breathing difficulties, chest pain, cough, sputum production, and fever [3].

Pneumonia can be classified in different ways. It may be defined as viral, bacterial, fungal, or parasitic pneumonia based on the causative pathogen. Based on the mode of acquisition, pneumonia can be classified as hospital-acquired pneumonia (HAP) or community-acquired pneumonia (CAP) [4].

Community-acquired atypical pneumonia is caused by bacteria, including *Mycoplasma pneumoniae*, *Chlamydia pneumoniae*, and *Legionella pneumophila,* representing 14% of all pneumonia cases worldwide [5]. These bacteria can cause atypical pneumonia of various severities ranging from mild, self-limiting infections to moderate cases; 25% of patients may require hospitalization, and 1% of patients require intensive care while the mortality rates can reach up to 5% of patients [6,7,8].

The reported incidence of community-acquired atypical pneumonia has increased in the last two decades in different parts of the world. For example, in the United States and Europe, the prevalence of the associated pathogens jumped from 6% to 40%. In China, Tao and colleagues noted excessive reporting of community-acquired atypical pneumonia caused by *M. pneumoniae* compared to community-acquired typical pneumonia commonly caused by *Streptococcus pneumoniae* and *Haemophilus influenzae* [9,10]. However, in many other countries, the poor implementation of testing for atypical pathogen hospitalized patients and the community has made it challenging to obtain accurate information on prevalence rates [11]. 

In Saudi Arabia, early observations reported the incidence of CAP caused by different bacterial agents such as *S. pneumoniae*, *M. pneumoniae*, *C. pneumoniae*, and *L. pneumophila* among hospitalized patients [12,13]. A recent nationwide retrospective analysis noted a remarkable lack of detecting CAP cases’ etiological agents [14]. Another eight years of observation on 800 hospitalized patients with pneumonia found that the proportion of CAP cases was much higher than that of HAP cases. In contrast, the rate of complications was lower among CAP patients than among HAP patients [15].

Most previous studies have been conducted on hospitalized patients, and there is limited knowledge about the prevalence and serostatus of atypical pneumonia-causing bacteria (*M. pneumoniae*, *C. pneumoniae*, and *L. pneumophila*) in the community. Therefore, we aimed in this study to investigate the seroprevalence of these bacterial agents among healthy individuals in the community by subjecting their sera to IgG sero-detection.

## 2. Methodology

### 2.1. Study Design and Participant Samples and Data

In this retrospective study, data and serum samples from 283 healthy individuals collected for previous projects [16,17] were used. Male participants (n = 144) were blood donors at the central blood bank in Medina province. Female participants (n = 139) were routine visitors to the women’s hospital and gynecology clinic in King Salman City in Medina province. No participants had any complaints of respiratory infections when obtaining the blood samples. Archived stored sera were preserved at −80 °C before thawing and processed for IgG sero-detection for *C. pneumoniae*, *L. pneumophila,* and *M. pneumoniae*. Patients’ data included age and gender. Ethical approval for this study was obtained from the Research Ethics Committee of the College of Applied Medical Sciences, Taibah University (2022/140/124/MLT). 

### 2.2. Serological Detection

#### 2.2.1. Indirect ELISA for *C. pneumoniae* and *L. pneumophila*

*C. pneumoniae* and *L. pneumophila* IgG antibodies were assessed using Serion ELISA classic kits for *C. pneumoniae* (ESR1371G, sensitivity 98.1%, specificity 96%; Institut VirionSerion GmbH, Würzburg, Germany) and *L. pneumophila* serogroups 1–7 (ESR106G, sensitivity 94.3%, specificity 86.5%; Institut VirionSerion GmbH, Würzburg, Germany) according to the protocol described. Samples were diluted at 1:100 for the *L. pneumophila* assay and 1:1500 for the *C. pneumoniae* assay with dilution buffer. The substrate blank was placed in a single well, and the standard and cut-off sera were duplicated. For 60 minutes, serum samples and control sera were incubated in microplates. After washing the wells, the IgG conjugate was added, and samples were incubated at 37 °C for 30 minutes. After the second round of washing, the substrate para-nitrophenylphosphate (pNPP) was added, and samples were incubated for 30 minutes. The absorbance at 405 nm was measured immediately after the stop solution was added to all wells. The upper and lower cut-off values or IgG concentrations (U/mL) were calculated using the quality control certificates included with the kits. All washing procedures were carried out by the manufacturer’s instructions using a semi-automated ELISA washer and reader (Biotek, Winooski, Chittenden County, Vermont, US).

#### 2.2.2. Indirect Enzyme-Linked Immunosorbent Assay (ELISA) for *M. pneumoniae*

The serum samples were also tested for *M. pneumoniae* IgG antibodies using Vircell IgG ELISA kits having a sensitivity of 98% and a specificity of 97% (G/M1015, Vircell, Granada, Spain) according to the manufacturer’s instructions. Briefly, serum samples and controls were diluted with sample diluent before incubating at 37 °C for 45 minutes. After the wells of the plate were washed, the IgG conjugate was added, and samples were incubated for 30 minutes at 37 °C. After another round of washing, the substrate tetramethylbenzidine (TMB) was added, incubating for 20 minutes at room temperature, and protected from light. Following the addition of the stop solution, the absorbance at 450/620 nm was measured. According to the manufacturer, antibody index values were obtained by dividing the sample optical density values by the means of the cut-off controls’ optical density values and multiplying by 10. 

### 2.3. Statistical Analyses

Fisher’s exact test was used to verify the association between two categorical variables (2 × 2 contingency tables, i.e., IgG seropositivity and different genders). A chi-square was conducted to investigate the distribution of IgG positivity among different age groups. In addition, Mann–Whitney U test was used to compare the median age for different participant groups that showed different patterns of seropositivity overlap. *p*-values ≤ 0.05 were considered statistically significant. Data were analyzed using the Statistical Package for the Social Sciences version 20 (IBM Corporation, Armonk, NY, USA).

## 3. Results

### 3.1. Demographic Information

Serums from 283 healthy individuals were collected in this work. Male participants comprised 50.9% of the sample population, and female subjects included 49.1%. The participants ranged from 18 to 72 years, with a mean age of 36.37 ± 11.21. There was no significant difference (*p* = 0.254) in age between men (37.49 ± 11.53 years) and women (35.22 ± 10.79 years). Participants were grouped by age (Table 1).

### 3.2. C. pneumoniae IgG Seropositivity

**Figure 1:**
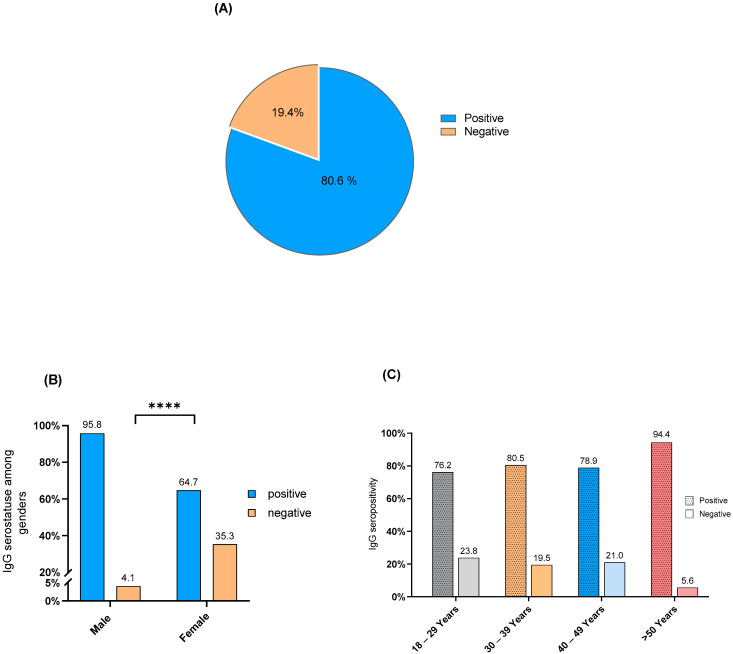
*C. pneumoniae* IgG seropositivity. (**A**) Seroprevalence in the sample population, (**B**) proportions of IgG seropositivity in different genders, (**C**) distribution of IgG seropositivity for different age groups. (****) *p*-value < 0.0001.

A remarkable level of *C. pneumoniae* IgG seropositivity rate was detected among the participants reaching 80.6% (228/283) (Figure 1A). In addition, *C. pneumoniae* IgG seropositivity occurred in 95.8% of the males and 64.7% of the females. Fisher’s exact test showed a significant association between IgG seropositivity and gender (*p*-value < 0.0001), and the majority of *C. pneumoniae* IgG seropositivity occurred among male participants (Figure 1B). Furthermore, chi-square analysis showed no significant difference in IgG seropositivity distribution between age groups (*p*-value = 0.133). However, *C. pneumoniae* IgG seropositivity exceeded two-thirds in each age group as it occurred in 76.2% of the participants in the age group 18–29 years, 80.5% in the age group 30–39 years, 78.9% in the age group 40–49, and 94.5% in the age group > 50 years (Figure 1C). 

### 3.3. L. pneumophila IgG Seropositivity 

**Figure 2:**
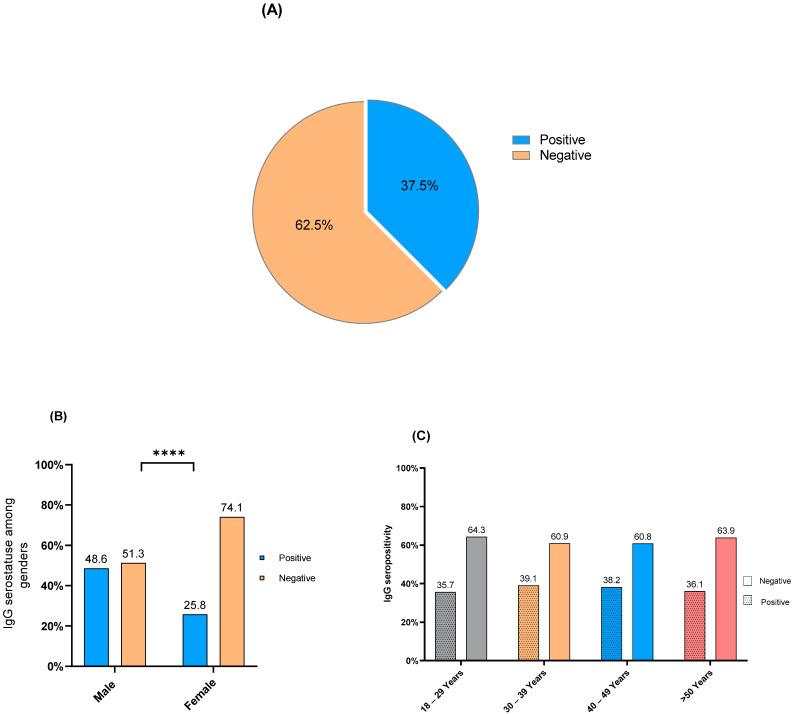
*L. pneumophila* IgG seropositivity in the community. (**A**) Seroprevalence in the sample population, (**B**) proportions of IgG seropositivity in different genders, (**C**) distribution of IgG seropositivity for different age groups. (****) *p*-value < 0.0001.

*L. pneumophila* IgG antibody detection revealed seropositivity in 37.5% of the sample population (106/283) (Figure 2A). In addition, *L. pneumophila* IgG seropositivity occurred in 48.6% of the males and 25.8% of the females. Fisher’s exact test showed a significant association between *L. pneumophila* IgG seropositivity and gender (*p*-value < 0.0001) and the most of *L. pneumophila* IgG seropositivity occurred among male participants (Figure 2B). Furthermore, chi-square analysis showed no significant difference in IgG seropositivity distribution between age groups (*p*-value = 0.978). However, *L. pneumophila* IgG seropositivity reached one third in each age group as it occurred in 35.3% of the participants in the age group 18–29 years, 39.1% in the age group 30–39 years, 38.2% in the age group 40–49, and 36.1% in the age group > 50 years (Figure 2C).

### 3.4. M. pneumoniae IgG Seropositivity 

**Figure 3:**
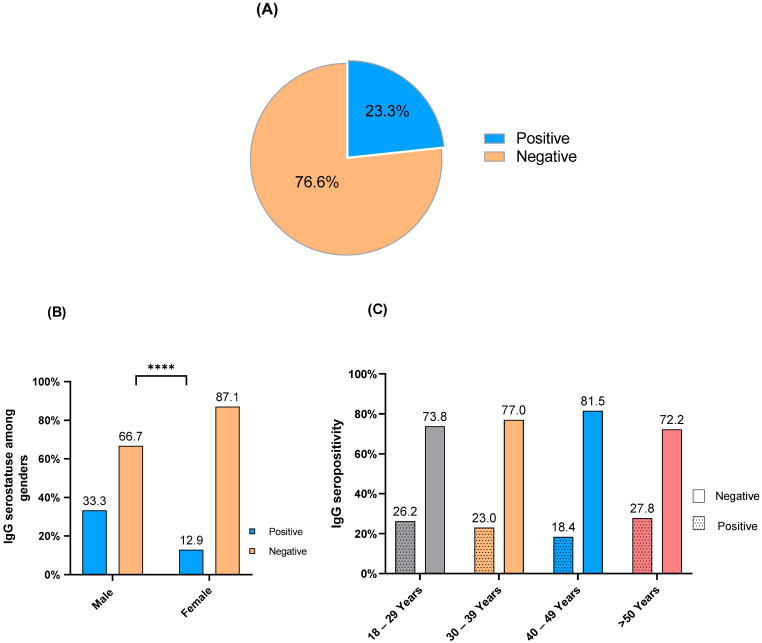
*M. pneumoniae* IgG seropositivity in the community. (**A**) Seroprevalence in the sample population, (**B**) proportions of IgG seropositivity in different genders, (**C**) distribution of IgG seropositivity for different age groups. (****) *p*-value < 0.0001.

Out of the 283 serum samples evaluated, *Mycoplasma* IgG seropositivity was detected in 66 (23.3%) of the samples (Figure 3A). In addition, *Mycoplasma* IgG seropositivity occurred in 33.3% of the male participants and in 12.9% of the females. Fisher’s exact test showed a significant association between *Mycoplasma* IgG seropositivity and gender (*p*-value < 0.0001), and the most of *Mycoplasma* IgG seropositivity appeared among male participants (Figure 3B). Moreover, *Mycoplasma* IgG seropositivity was observed in 26.2% of the participants in the age group 18–29 years, 23% in the age group 30–39 years, 18.4% in the age group 40–49 years, and 27.8% in age group > 50 years (Figure 3C). There was no difference in the distribution of *Mycoplasma* IgG seropositivity among age groups (chi-square 1.81, *p*-value = 0.612).

### 3.5. IgG seropositivity and overlapping 

**Figure 4:**
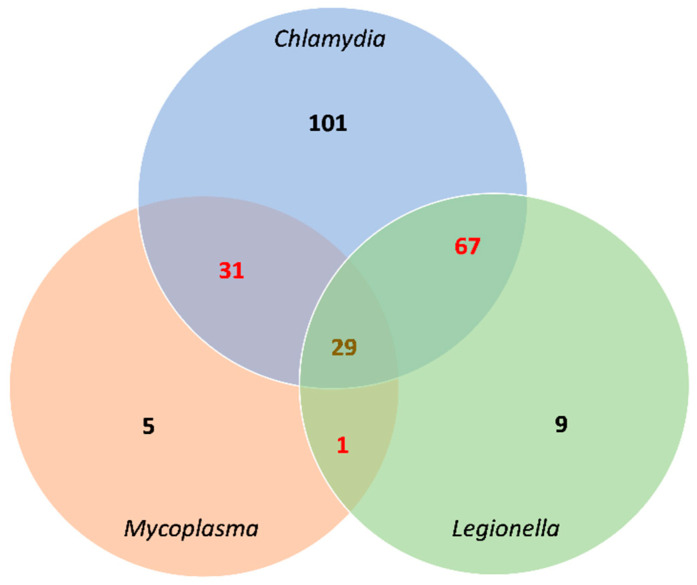
Venn diagram shows the overlapping of IgG seropositivity results between different bacterial species. **Black** numbers for IgG seropositivity without overlapping with other species. **Red** numbers for IgG seropositivity with overlapping between two species. **Brown** number for IgG seropositivity with overlapping between three species.

The total of IgG seropositivity for the three causative atypical pneumonia bacteria was detected in 85.8% (243/283) of the sample population studied here. In addition, seropositivity appeared in three patterns; the first was seropositivity for only one bacterial species that was noted in 115 out of 283 (40.6%) samples. The second was IgG seropositivity for two species of bacteria which was reported in 99 (35%) samples. The third pattern was seropositivity detected for three species and occurred in 29 (10.2%) of the screened samples (Figure 4).

In addition, the **Mann–Whitney test** revealed no significant difference in the median of age for participants that showed seropositivity for one organism and those that showed seropositivity with two organisms (*p*-value 0.5967) or three organisms (*p*-value 0.3859). Moreover, comparing between participants that showed seropositivity with two organisms and those who showed seropositivity with three organisms, no significant difference in the median age occurred between them (*p*-value 0.2496). 

**Figure 5:**
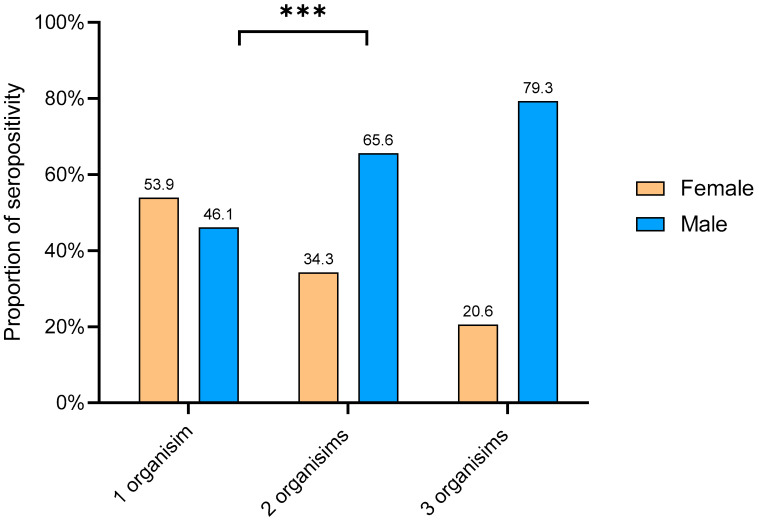
Distribution of IgG seropositivity among male and female participants in the different seropositivity groups. (***) *p*-value = 0.0007.

Furthermore, seropositivity patterns between different genders were observed and illustrated in Figure 5. The chi-square test showed a significant association between genders and IgG seropositivity patterns (chi-square 14.5, *p*-value = 0.0007), and IgG seropositivity patterns appeared more commonly among male participants compared to females (Table 2).

## 4. Discussion

This retrospective study investigated the prevalence of IgG seropositivity for *C. pneumoniae*, *L. pneumophila*, and *M. pneumoniae* in 283 healthy participants to determine the serostatus of these community-acquired atypical pneumonia-causing bacteria in Medina. The IgG seropositivity for these bacteria indicates previous incidence(s) of infections that may have persisted for months and years because of re-infection [18,19]. The results revealed the high distribution of IgG seropositivity for these bacterial species among healthy individuals.

For instance, *C. pneumoniae* IgG seropositivity was detected in 80% of the sample population, which agrees with previous observations in different countries where the prevalence ranged from 40–86 % [20,21,22]. However, some countries like Germany and Norway reported a lower prevalence of *C. pneumoniae* (0.9% and 3%, respectively) [23,24]. In addition, IgG sero-screening for *C. pneumoniae* showed a predominance of seropositivity among men (Figure 1B), which is congruent with the recent work conducted by Al-Aydie and colleagues [25]; it is unclear whether specific factors influence the infectivity in different genders. Furthermore, no significant difference in IgG seropositivity distribution occurred between age groups, which is consistent with other researchers’ results [26]. We noticed that C. pneumoniae IgG seropositivity was detected at least in 75% of the participants in each age group, including young adults, such as those at the age of 18–39 years old (Figure 1C). Previous work by Al-Younes [22] showed a predominance of *C. pneumoniae* IgG seropositivity among healthy participants in their twenties and thirties. Separate epidemiological area and population target groups, as well as variations in the diagnostic techniques used in these studies, may have led to these contrary findings. 

*L. pneumophila* IgG seropositivity occurred in 37.5% of the sample population. Similar to the *C. pneumoniae* results, *L. pneumophila* IgG sero-detection revealed predominant positivity among men, which aligns with recent observations [27]. In addition, in each age group, one-third of the participants showed IgG positivity with *L. pneumophila* and there was no significant difference in IgG seropositivity distribution between different age groups (Figure 2C). Water supply is a potential source for *L. pneumophila* infections. Recent studies in Saudi Arabia showed that 11% of the samples collected from hotel water tanks were positive for the bacterium [28]. We assume that water contamination with *L. pneumophila* and inadequate quality control policies may have caused the high level of IgG seropositivity in the community. Nevertheless, growing rates of *L. pneumophila* incidence have been noted globally in past decades, which can be attributed to the increasing awareness of the disease, the emergence of the diversity for *L. pneumophila* diagnosis, and the spreading of the bacteria from environmental sources such as cooling systems, air conditioners, baths, and showers [29].

*M. pneumoniae* IgG seropositivity occurred in 23.3% of the sample population, concordant with other observations [30]. In addition, *M. pneumoniae* seropositivity was more common among male participants than females (Figure 3B), which corresponded with previous observations in Saudi Arabia [31]. In addition, M. pneumoniae seroprevalence showed no significant difference in distribution between different age groups. However, other researchers [32,33] reported significant differences in the distribution of the bacterium among children who were not included in our sample population. 

Overlapping of IgG seropositivity for *C. pneumoniae, L. pneumophila*, and *M. pneumoniae* was noted in 85.8% (243/283) of the healthy participants (Figure 4) and there was no significant difference in participants’ median age between different seropositive groups. However, there was a significant association between genders and IgG seropositivity patterns as IgG seropositivity patterns appeared more commonly among male participants compared to females. It is unclear if this overlap indicates simultaneous infections with these pathogens or not. Concurrent infections or co-infections of these atypical pneumonia-causing bacteria were reported among hospitalized patients [26,34]. It is unknown whether there is a linking factor that facilitates co-infections with these organisms or if infection with one species paves the way for the others. A point of co-infection that is yet to be elucidated and understood. However, we assume participants had previous infections with those bacteria during their lives and the persistence of IgG antibodies.

Several studies have stated the clinical importance of these bacterial infections as they can cause more severe infection than ordinary; for example, *C. pneumoniae* infections have been associated with other serious diseases such as coronary heart disease, atherosclerosis, lung cancer, and asthma [35,36,37,38]. Furthermore, in some cases, *M. pneumoniae* infections may lead to other complications such as polyarthritis, neurological diseases, hemolytic anemia, and erythema multiform [39]. Moreover, a French retrospective multicenter study reported that extrapulmonary complications occurred in one-third of atypical pneumonia patients admitted to the intensive care unit [40]. 

It is crucial to remember that certain groups in the community are more susceptible to atypical pneumonia and its complications. These include smokers, the elderly, individuals with chronic diseases such as diabetes mellitus, liver, and renal disease, cancer and cardiovascular disease, and immunosuppressed patients [41,42,43]. Therefore, the high seroprevalence of atypical pneumonia-causing bacteria in the community reported here, along with the unavailability of vaccines against these bacterial species, indicates the requirement for implementing prevention strategies in both community and healthcare service settings to keep the possibility of their clinical complications at a minimum rate. These strategies should include tracking the incidence of *M. pneumoniae*, *C. pneumoniae*, and *L. pneumophila* and determining antibiotic susceptibility. In addition, monitoring and maintaining water systems and devices at risk for *Legionella* growth is important to prevent them from becoming potential sources of infection. 

## 5. Limitations of the Study

Our work has several limitations. As this was a retrospective study, we could not collect certain participants’ information, such as physical activity status, smoking habits, and chronic disease, even though these are risk factors for atypical pneumonia infections. We could not determine whether the participants had previously suffered from atypical pneumonia and had been treated. Moreover, knowledge of whether participants had been hospitalized would have allowed the exclusion of their sero-response from the category of community-acquired infection. However, we shed light on the seroprevalence of *C. pneumoniae*, *L. pneumophila*, and *M. pneumoniae* among healthy individuals in the community, which has not been done previously. 

## 6. Concluding Remarks

In conclusion, our work documented a high distribution of IgG seropositivity for *C. pneumoniae*, *L. pneumophila*, and *M. pneumoniae* among healthy individuals, indicating a raised prevalence of these bacteria in the community. In addition, their incidences among healthy individuals tend to be expected in young adult groups rather than the elderly and occur more among males than females.

Improving preventive programs and policies against these bacteria is essential to avoid the clinical complications of infection among susceptible groups in the community. It may even lead to reduced rates of hospitalization.

## Figures and Tables

**Table 1:** Demographic characteristics for study participants (n = 283).

Categories	N (%)
Gender	
Male	144 (50.9)
Female	139 (49.1)
Total	283
Age groups	
18–29	84 (29.7)
30–39	87 (30.7)
40–49	76 (26.9)
>50	36 (12.7)
Total	283

**Table 2:** Association between seropositivity overlap groups and genders.

Seropositivity Overlap Groups	N (%)		Total	*p*-Value
	Male	Female		
One organism	53 (46.1%)	62 (53.9 %)	115	0.0007
Two organisms	65 (65.6 %)	34 (34.3%)	99	
Three organisms	23 (79.3%)	6 (20.6%)	29	
Total	141 (58%)	102 (42%)	**243**	

## Data Availability

All data and materials generated during the current study are available from the corresponding author upon reasonable request.
